# Supplementary material for: Life history shapes variation in egg composition in the blue tit Cyanistes caeruleus
Source: Commun Biol. 2019 Jan 4;2:6. doi: 10.1038/s42003-018-0247-8 (PMC6320336; doi:10.1038/s42003-018-0247-8)
Supplement: Supplementary file 1 — Description of Additional Supplementary Files [file 42003_2018_247_MOESM1_ESM.docx]

**Description of Additional Supplementary Files**

**File Name**: Supplementary Data 1

**Description**: **Predictors of egg composition.**

Results of linear mixed-effect models with laying date, clutch size, female age and laying order as predictors. Female identity and laying order were included as random intercept and slope, respectively. Carotenoid concentrations were log transformed. Female age estimates are for adults relative to yearlings, embryo sex estimates are for males relative to females, paternity estimates are for extra-pair sired eggs relative to within-pair. Model formula: (reference model) *Dependent variable ~ Laying date + Clutch size + Female age + Laying order + (Laying order|Female ID).* Models with paternity as predictor are applied to first eggs of the clutches: *Dependent variable ~ Paternity + Laying date + Clutch size + Female age + (Laying order|Female ID).* Significant negative estimates are in blue, significant positive estimates are in red.

**File Name**: Supplementary Data 2

**Description**: **List of proteins identified in the blue tit egg albumen and yolk.**

Technical descriptors for all egg proteins identified. PID: protein group identifier; Protein ID: identifier for the proteins in the protein group; Reference sequence: RefSeq identifier in the NCBI Reference Sequence Database; Best homologue; Protein short name; Species: species in which the best homologue was found; Gene ID: NCBI gene identifier; Gene name; No. of proteins: number of proteins in the group; Peptides: the total number of peptide sequences associated with the protein group (i.e. for all the proteins in the group); Razor + unique peptides: the total number of razor and unique peptides associated with the protein group (i.e. these peptides are shared with another protein group); Unique peptides: the total number of unique peptides associated with the protein group (i.e. these peptides are not shared with another protein group); Sequence coverage: percentage of the sequence that is covered by the identified peptides of the best protein sequence contained in the group; No. of nests in which the protein was identified; No. of eggs in which the protein was identified; Functional classification of the proteins according to the Functional Classification Catalogue Version 2.1: given are the FunCat term and the corresponding FunCat number.

**File Name**: Supplementary Data 3

**Description**: **List of proteins quantitated in the blue tit egg albumen and yolk, their predictors and correlations with yolk lipids and carotenoids.**

Technical descriptors for all egg proteins quantitated. PID: protein group identifier; Protein short name; Best homologue; Gene ID: NCBI gene identifier; Corresponding PID: the PID identifier of the protein in the other egg compartment; No. of nests in which the protein was identified; No. of eggs in which the protein was identified; Protein abundance (μg); Protein concentration (μg / g wet tissue); Model details: for each predictor, given are the estimate, SE, z value, adjusted p value; Protein Cluster ID: identifier of the cluster to which the protein was assigned by affinity propagation clustering (Supplementary Data 4).

Results shown are from linear mixed-effect models with the rank-transformed, normalized protein concentration as the dependent variable and laying date, clutch size, female age, embryo sex and laying order as predictors. Female identity and MS analysis batch were included as random intercepts and laying order as random slope. Female age estimates are for adults relative to yearlings, embryo sex estimates are for males relative to females, paternity estimates are for extra-pair sired eggs relative to within-pair. Model formula: (reference model) *Protein concentration ~ Egg compartment weight + Laying date + Clutch size + Female age + Laying order + Embryo sex + (Laying order|Female ID) + (1|MS batch).* Models with paternity as predictor are applied to first eggs of the clutches: *Protein concentration ~ Paternity + Egg compartment weight + Laying date + Clutch size + Female age + Embryo sex + (Laying order|Female ID) + (1|MS batch).* Significant negative estimates are blue, significant positive estimates are red.

**File Name**: Supplementary Data 4

**Description**: **Functional classes of proteins in the blue tit egg albumen and yolk.**

Characterisation of the functional categories of proteins based on Gene Ontology and the MIPS Functional Catalogue database. N identified: the number of proteins in albumen and yolk that fit each functional category, whereby one protein can be assigned to multiple categories; Specificity: the egg compartment where a protein has been identified (albumen, yolk or both); Concentration: the mean and standard deviation of the cumulative concentration of all proteins that belong to a certain functional category.

**File Name**: Supplementary Data 5

**Description**: **Predictors of egg weight; egg compartment allometry.**

Predictors of egg weight: results of linear mixed-effect models with laying date, clutch size, female age and laying order as predictors. Female identity and laying order were included as random intercept and slope, respectively. Female age estimates are for adults relative to yearlings, embryo sex estimates are for males relative to females, paternity estimates are for extra-pair sired eggs relative to within-pair. Model formula: (reference model) *Egg weight ~ Laying date + Clutch size + Female age + Laying order + (Laying order|Female ID).* Models with paternity as predictor are applied to first eggs of the clutches: *Egg weight ~ Paternity + Laying date + Clutch size + Female age + Laying order|Female ID).* Significant negative estimates are in blue, significant positive estimates are in red.

Egg compartment allometry: results from linear mixed-effect models with the egg compartment weight relative to egg weight as dependent variable, egg weight as predictor, female identity as random intercept and egg weight as a random slope. Model formula: C*ompartment weight/Egg weight ~ Egg weight + (Egg weight|Female ID)*. Significant negative estimates are in blue, significant positive estimates are in red.

**File Name**: Supplementary Data 6

**Description**: **Predictors of measures of reproductive success in the population (2007-2015) and during the 2014 breeding season.**

Shown are results of generalised linear models. Models with clutch size and laying date as dependent variable are ordinary least-squares models, while those with hatching and fledging success as dependent variable have a binomial error distribution. Multiple-year models were linear mixed-effect models with year as random factor. Laying date was centred for each year by subtracting the year's median. Female age estimates are for adults relative to yearlings. Significant negative estimates are in blue, significant positive estimates are in red.

**File Name**: Supplementary Data 7

**Description**: **Predictors of embryo sex and extra-pair paternity.**

For embryo sex, the results are from a generalized linear mixed-effect model with a binomial error distribution with embryo sex (1: male, 0: female) as dependent variable and with laying date, clutch size, female age and laying order as predictors. Female identity and laying order were included as random intercept and slope, respectively. Female age estimates are for adults relative to yearlings. Model formula: *Embryo sex ~ Laying date + Clutch size + Female age + Laying order + (Laying order|Female ID), family = binomial.* For extra-pair paternity, results are from a generalized linear model with a binomial error distribution with paternity (1: extra-pair, 0: within-pair) as dependent variable and with laying date, clutch size, female age and laying order as predictors. Female age estimates are for adults relative to yearlings, embryo sex estimates are for males relative to females. Model formula: *Paternity ~ Laying date + Clutch size + Female age + Embryo sex, family = binomial.*

**File Name**: Supplementary Data 8

**Description**: **Predictors of the concentration and relative abundance of the functional classes of proteins.**

Results of linear mixed-effect models for each FunCat term and egg compartment with the sum of the protein concentrations per class (rank-transformed) as dependent variable and laying date, clutch size, female age, embryo sex and laying order as predictors. Female identity and laying order were included as random intercept and slope, respectively. MS batch was also included as random intercept. Female age estimates are for adults relative to yearlings, embryo sex estimates are for males relative to females, paternity estimates are for extra-pair sired eggs relative to within-pair. Model formula: (reference model) *FunCat concentration (relative abundance) ~ Laying date + Clutch size + Female age + Embryo sex + Laying order + (Laying order|Female ID) + (1|MS batch).* Models with paternity as predictor are applied to first eggs of the clutches: *FunCat concentration (relative abundance) ~ Paternity + Laying date + Clutch size + Female age + Embryo sex + (Laying order|Female ID) + (1|MS batch).*  Significant negative estimates are in blue, significant positive estimates are in red.

**File Name**: Supplementary Data 9

**Description**: **Predictors of the concentration of the albumen and yolk protein clusters.**

Results from linear mixed-effect models for each protein cluster with the sum of the protein concentrations per cluster (rank-transformed) as dependent variable and with laying date, clutch size, female age, embryo sex and laying order as predictors. Female identity and laying order were included as random intercept and slope, respectively. MS batch was also included as random intercept. Female age estimates are for adults relative to yearlings, embryo sex estimates are for males relative to females, paternity estimates are for extra-pair sired eggs relative to within-pair. Model formula: (reference model) *Protein cluster concentration ~ Egg compartment weight + Laying date + Clutch size + Female age + Embryo sex + Laying order + (Laying order|Female ID) + (1|MS batch).* Models with paternity as predictor are applied to first eggs of the clutches: *Protein cluster concentration ~ Paternity + Egg compartment weight + Laying date + Clutch size + Female age + Embryo sex + (Laying order|Female ID) + (1|MS batch).* Significant negative estimates are in blue, significant positive estimates are in red.

**File Name**: Supplementary Data 10

**Description**: **Predictors of the individual yolk carotenoid concentrations.**

Shown are abundance and concentration of the individual carotenoids and the results of linear mixed-effect models for each carotenoid with the log-transformed concentration as the dependent variable and laying date, clutch size, female age, embryo sex and laying order as predictors. Female identity and laying order were included as random intercept and slope, respectively. HPLC analysis batch was also included as random intercept. Female age estimates are for adults relative to yearlings, embryo sex estimates are for males relative to females, paternity estimates are for extra-pair sired eggs relative to within-pair. Model formula: (reference model) *log(Carotenoid concentration) ~ Egg compartment weight + Laying date + Clutch size + Female age + Laying order + Embryo sex + (Laying order|Female ID) + (1|HPLC batch).* Models with paternity as predictor are applied to first eggs of the clutches: *log(Carotenoid concentration) ~ Paternity + Egg compartment weight + Laying date + Clutch size + Female age + Embryo sex + (Laying order|Female ID) + (1|HPLC batch).* Significant negative estimates are in blue, significant positive estimates are in red.

**File Name**: Supplementary Data 11

**Description**: **Description of the chemical composition of the egg clusters.**

The composition (albumen and yolk protein clusters concentrations, lipid concentration, total carotenoids concentration and egg weight) of the three egg clusters was compared using ANOVA. Post-hoc tests of significant differences (after false discovery rate correction) are shown in Fig. 6d.

**File Name**: Supplementary Data 12

**Description**: **Predictors of the egg clusters.**

Shown are the results of functional chi-square tests used to determine whether clutch size, female age, laying date, laying order, embryo sex and paternity predicted egg assignment to a given clusters. Clutch size, laying date and laying order were categorised into 3 levels (low, average and high) based on equal intervals.

**File Name**: Supplementary Data 13

**Description**: **Description of sampled nests.**

Description of sampled nests compared to all nests during the same breeding season (2014) and across all breeding seasons (2007-2015) in the Westerholz blue tit population.
