# Supplementary material for: Life history shapes variation in egg composition in the blue tit Cyanistes caeruleus
Source: Commun Biol. 2019 Jan 4;2:6. doi: 10.1038/s42003-018-0247-8 (PMC6320336; doi:10.1038/s42003-018-0247-8)
Supplement: Supplementary file 2 — Supplementary Information [file 42003_2018_247_MOESM2_ESM.pdf]

SUPPLEMENTARY NOTES

1. Breeding conditions in 2014 compared to other years

The breeding season of 2014 was the earliest and shortest season in 9 years of observations (Supplementary Data 13). In this season, relatively few pairs bred and females laid the highest mean clutch size with the lowest variance recorded in this population. The proportion of adult birds breeding in 2014 was the second highest and the percentage of male offspring and fledging success were the highest recorded (Supplementary Data 13). The early start and short duration of the 2014 breeding season together with the low breeding density may explain the high fledging success recorded in this year. Typically, in blue tits, early broods have heavier young[1] with higher survival[2,3] and recruitment chances[1] due to a better coordination with the maximum caterpillar supply[3,4] and/or to a competitive advantage during the early fledgling stage.

Between years, mean clutch size decreased with the number of breeding pairs ($r=-0.84$, $N=9$, $p=0.004$) and with the onset of the breeding season (date of first egg in the population: $r = -0.69$, $N=9$, $p=0.041$). As observed in other populations[1,5-11], clutch size decreased within season on average with 0.13 [0.11,0.15] eggs/day (mean, 95% CI), independently of female age (Supplementary Data 6). The decrease in clutch size with laying date (date of the first egg of the clutch) was somewhat lower in 2014 than in the other years (0.10 [0.04,0.16] eggs/day) (Supplementary Data 6). Adult females started laying earlier than yearling females in all years (as is typical in other tit populations[2]), but not in 2014 (Supplementary Data 6), perhaps because the breeding season was so short and synchronous. Hatching and fledging success were independent of clutch size, female age and laying date in all years (Supplementary Data 6).

2. Predictors of variation in the concentration of defence and immunity-related egg proteins

Birds deposit antimicrobial proteins in both the yolk and the albumen. Antimicrobial proteins in the yolk reach the embryonic blood and protect the embryo from systemic infections, while antimicrobials in the albumen are ingested with the rest of the albumen before hatching and may play a more important role in the local intestinal protection of the hatchling[12]. Because of their importance for offspring survival, previous studies on protein allocation to avian eggs predominantly addressed the following four defence and immunity-related proteins.

Avidin is a biotin-sequestering protein[13], which inhibits the growth of biotin-requiring microorganisms such as bacteria and yeast[14]. A study on six passerine species found that avidin levels in the albumen increased with laying order in two of them (violet-green swallows *Tachycineta thalassina* and tree swallows *Tachycineta bicolor*)[15], but so far no differential deposition of avidin has been found within blue tit clutches[16]. We identified an avidin-like protein (yp192) in the yolk and an uncharacterized protein similar to avidin (ap226, yp191) in the yolk and the albumen. The concentration of the albumen protein ap226 increased with laying order (Supplementary Data 3). None of the other tested variables explained variation in the concentrations of the yolk avidin isoforms (all p values > 0.99).

Ovotransferrin is an acidic, iron-binding glycoprotein, which transports iron to the developing embryo. The protein has antibacterial, antifungal, antiviral, antioxidative and growth-promoting properties, mediated both by its iron-binding capacity and by specific bioactive peptides[17]. In our samples, ovotransferrin was the fourth most abundant albumen protein and the eighth most abundant yolk protein. A previous study on six passerine species found that ovotransferrin decreased with laying order in one of them (the pearly-eyed thrasher *Margarops fuscatus*[15]), while a study on blue tits found higher levels of ovotransferrin in the eggs in the middle of the laying sequence[16]. In our population, ovotransferrin (ap221, yp190) concentrations significantly increased

with laying order in both egg compartments (Supplementary Data 3). In addition, in the yolk, transferrin receptor protein 1 (yp89) also increased in concentration with laying order (Supplementary Data 3). Ovotransferrin concentration did not depend on any of the other predictors (Supplementary Data 3).

Lysozyme is a glycoside hydrolase, which hydrolyses peptidoglycans in the bacterial cell wall. The protein has bacteriolytic, antiviral and antioxidant activities[18]. Moreover, a recent study on domestic Tsaiya ducks (related to mallard *Anas platyrhynchos*) described an association between a SNP marker of the lysozyme gene and egg hatchability[19]. A recent study showed that, although egg shells have higher bacterial loads in great tits compared to blue tits, higher levels of lysozyme and immunoglobulins protect great tit eggs such that their hatching success is only marginally lower than in blue tit eggs[20]. Within species, lysozyme activity correlates negatively with egg weight in pied flycatchers *Ficedula hypoleuca*[21] and with clutch size in barn swallows *Hirundo rustica*[22]. Barn swallows also seem to deposit less lysozyme at lower environmental temperatures[23], suggesting that, at least in some species, deposition of lysozyme (or of proteins in general) is costly for females. Previous studies on several species found evidence that females deposit more albumen lysozyme when mated to high quality males. For example, the lysozyme concentration in the albumen correlated positively with male song complexity in Eurasian reed warblers *Acrocephalus scirpaceus*[24], with male bill coloration in mallards *Anas platyrhynchos*[25] and with male UV chroma in blue tits[16]. Blue tit females also laid eggs with higher concentrations of lysozyme when mated to monogamous males[16]. In our blue tit population, lysozyme (ap86, yp74) concentration did not vary with laying order or with any of the other predictors (Supplementary Data 3). The absence of a laying order effect confirms previous work on passerines[15], including blue tits[16]. Only one study on barn swallows showed a decrease of the albumen lysozyme concentration with laying order[22]. This study also showed that the lysozyme levels in the albumen correlated positively with lysozyme activity in 5-days old nestlings.

Immunoglobulins (Ig) are circulating glycoproteins produced by the immune system, which bind specific antigens and contribute to the defence against invading pathogens[26]. They are the most studied mediators of maternal effects in vertebrates[12]. Nestlings cannot produce immunoglobulins until several days after hatching so they rely on maternally transferred immunoglobulins[27]. Maternal immunoglobulins deposited in the egg confer passive protection to the developing embryo and can assist the development of the hatchling's immune system[12]. Birds predominantly deposit Ig A and Ig M in the albumen and Ig Y (homologue of the mammalian Ig G) in the yolk, although the yolk can also contain small amounts of Ig A and Ig M[26,28]. We identified and quantitated an Ig G homologue (yp100) and Ig M (yp83) in the egg yolk. Neither of them varied in concentration among eggs (Supplementary Data 3). We also identified and quantitated in both egg compartments a peptide representing the variable region of the heavy Ig chain (ap114, yp98). Its concentration did not vary in the yolk, but increased with laying order in the albumen (Supplementary Data 3). However, the albumen isoform was only quantified in 12 eggs from 9 nests, hence further investigation is required to confirm its variation. Immunoglobulin concentrations did not depend on any of the predictors (Supplementary Data 3). In other passerine species, females typically deposit higher concentrations of yolk immunoglobulins when they are in better condition[29], under higher predation risk[30], exposed to ectoparasites[31] or mated to more attractive males[32]. There are also some indications that females can differentially allocate yolk immunoglobulins within their clutch: in collared flycatchers *Ficedula albicollis*, the last-laid eggs contained higher concentrations[29], while in great tits concentrations increased with laying order (but only when females were experimentally exposed to parasites)[31]. A study on barn swallows suggested that yolk immunoglobulins are allocated differentially along the laying sequence depending on the attractiveness of the male[32]. Interestingly, in the magpie (*Pica pica*), nestlings with higher immunoglobulin levels survived better than their siblings, but nests with

high immunoglobulin levels had overall lower survival than nests with low immunoglobulin levels[33]. This is compatible with the notion that higher immunoglobulin levels reflect higher exposure to parasites and may explain why yolk immunoglobulin concentrations correlated negatively with fledging success of flycatchers broods[21].

3. Yolk carotenoids: function and sources of variation

Carotenoids are liposoluble, biologically active pigments, with antioxidant and immunomodulatory properties. The rapidly growing embryo is susceptible to oxidative stress due to the high rate of oxidative metabolism and to its high levels of polyunsaturated fatty acids, particularly at the time of hatching[34]. The carotenoids are part of a complex antioxidant system, which protects the yolk content and the developing embryo from oxidative damage caused by free radicals and toxic metabolic by-products. β-carotene and β-zeacarotene are precursors of vitamin A, they assist the recycling of other yolk antioxidants, stabilize the cell membrane, regulate cell proliferation and promote the maturation of the immune system[34]. Carotenoids cannot be synthesized *de novo* by animals and are obtained solely from the diet. Carotenoid composition varies between different prey species of blue and great tits[35,36]. The deposition of yolk carotenoids in their eggs is known to depend on habitat[37], laying date[38,39], laying order[37], yolk weight[39], female health[40], age[40], diet[41], and absorption and metabolising efficiency[42], and on the ornamentation of the male (male coloration[38,39]). Although their role in adult birds is still debated[43], egg carotenoids are believed to promote nestling growth[41,44], immune system development [41,45] and ability to incorporate plasma carotenoids into growing feathers[45].

SUPPLEMENTARY FIGURES

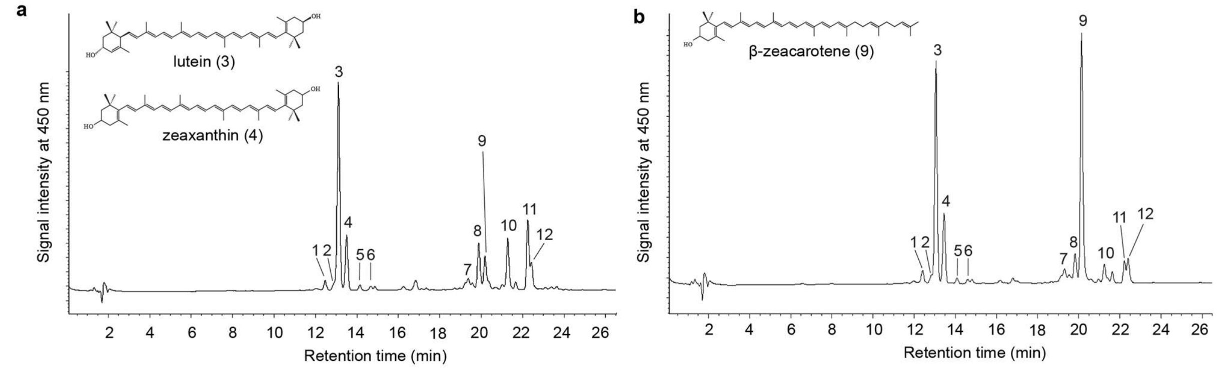

c

ID	Compound	Retention time LC-MS [min]	Retention time HPLC [min]	UV/Vis absorption maxima [nm]	HPLC/APCI-MS $[M]^-$ *m/z*	HPLC/APCI-MS $[M+H]^+$ *m/z*	Identification
1	(13Z)-lutein		12.5	n.a.	n.a.	n.a.	Tentative
2	(13Z)-zeaxanthin		12.9	n.a.	n.a.	n.a.	Tentative
3	lutein	13.9	13.1	422/446/474	568	569, 551*	Unambiguous
4	zeaxanthin	14.3	13.5	428/452/478	568	569	Unambiguous
5	(9Z)-lutein		14.2	n.a.	n.a.	n.a.	Tentative
6	(9Z)-zeaxanthin		14.7	n.a.	n.a.	n.a.	Tentative
7	α-carotene	19.87	19.4	422/446/474	536	537	Unambiguous
8	β-carotene	20.4	19.9	428/452/478	536	537	Unambiguous
9	β-zeacarotene	20.7	20.2	406/428/454	538	539	Unambiguous
10	unknown	21.9	21.3	422/444/472	538	539	Tentative
11	γ-carotene	22.99	22.3	438/462/492	536	537	Unambiguous
12	γ-carotene (Z)-isomer	23.12	22.4	438/462/492	536	537	Tentative

Supplementary Figure 1. Blue tit egg yolk carotenoids. a, b Chromatographic separation of carotenoids from blue tit egg yolk monitored at 450 nm. **a,** Common chromatogram. **b,** Chromatogram from an unusually β-zeacarotene-rich sample. Peak numbers and identification parameters correspond to compounds in panel c. **c,** Description of egg yolk carotenoids identified in yolk of blue tit eggs. HPLC retention time, UV/Vis absorption maxima, HPLC/APCI-MS $[M]^-$ *m/z*: mass-to-charge ratio for the molecular ion $[M]^-$, HPLC/APCI -MS $[M+H]^+$ *m/z*: mass-to-charge ratio for the protonated molecular ion $[M+H]^+$.

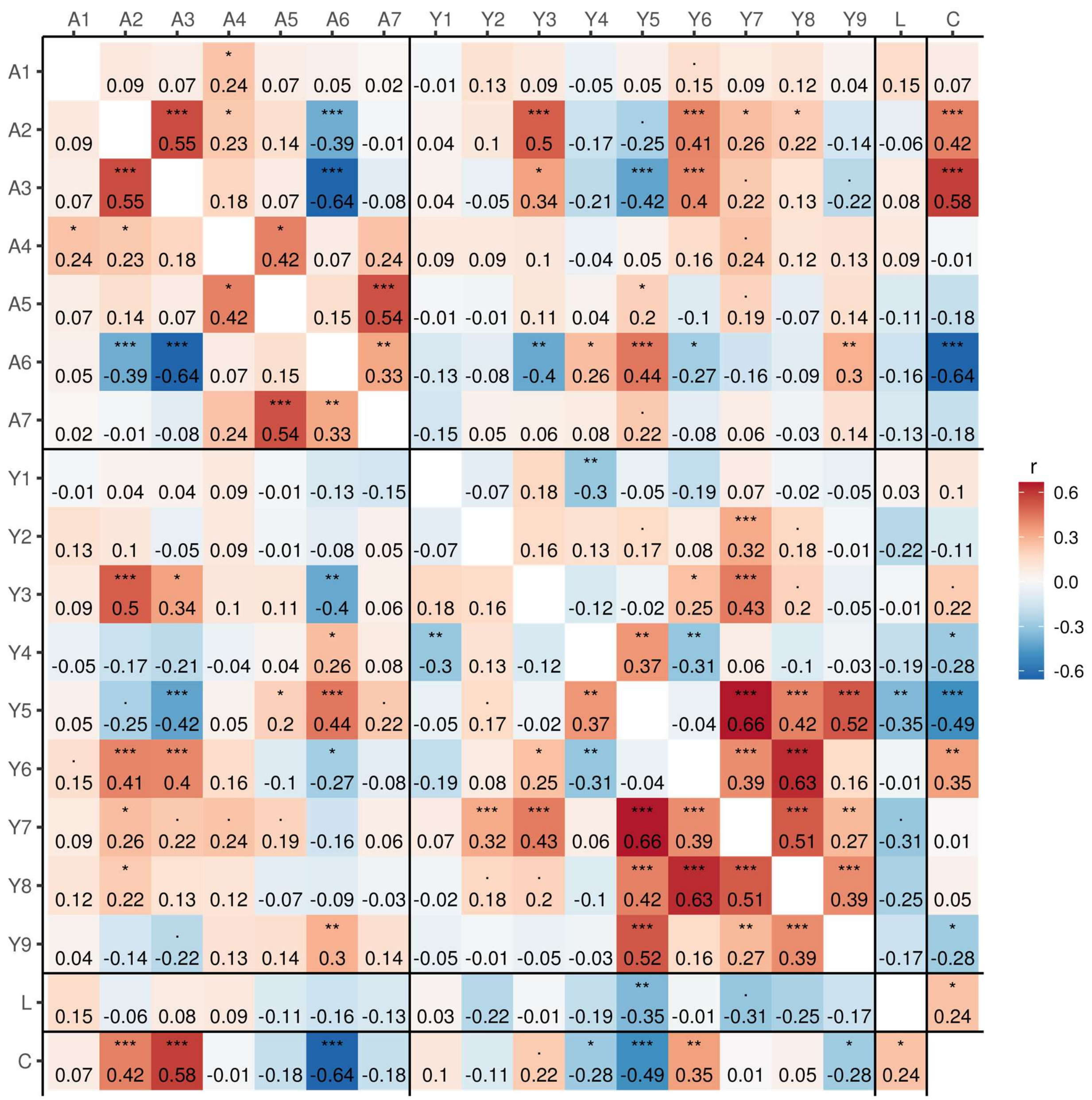

Supplementary Figure 2. Correlation matrix heat map for protein clusters, lipids and carotenoids in blue tit eggs. Shown are Pearson's r correlation coefficients between the concentrations of the albumen protein clusters (A1 – A7), yolk protein clusters (Y1 – Y9), yolk lipids (L) and yolk carotenoids (C). N = 109 eggs / 38 clutches. * P < 0.05, ** P < 0.01, *** P < 0.001. Cluster pairs A2 & A3, A5 & A7, A6 & A7, A2 & A6, A3 & A6, Y5 & Y9, Y4 & Y5, and Y4 & Y6 are significantly correlated possibly as a consequence of their similar variation with laying order. Cluster pairs Y5 & Y8, Y6 & Y7, Y6 & Y8, Y5 & Y7 and Y7 & Y8 are significantly correlated possibly as a consequence of their similar variation with paternity. Clusters Y1 & Y4 are negatively correlated, possibly due to their opposite (most NS) estimates for five of the six predictors (Supplementary Data 9). Clusters Y5 & Y6 are not correlated possibly because they vary similarly with paternity but oppositely with laying order. The significant positive correlations among cluster pairs A2 & A4, A4 & A5, Y2 & Y7, Y7 & Y9, Y8 & Y9, Y3 & Y7 cannot be explained based on the considered predictors.

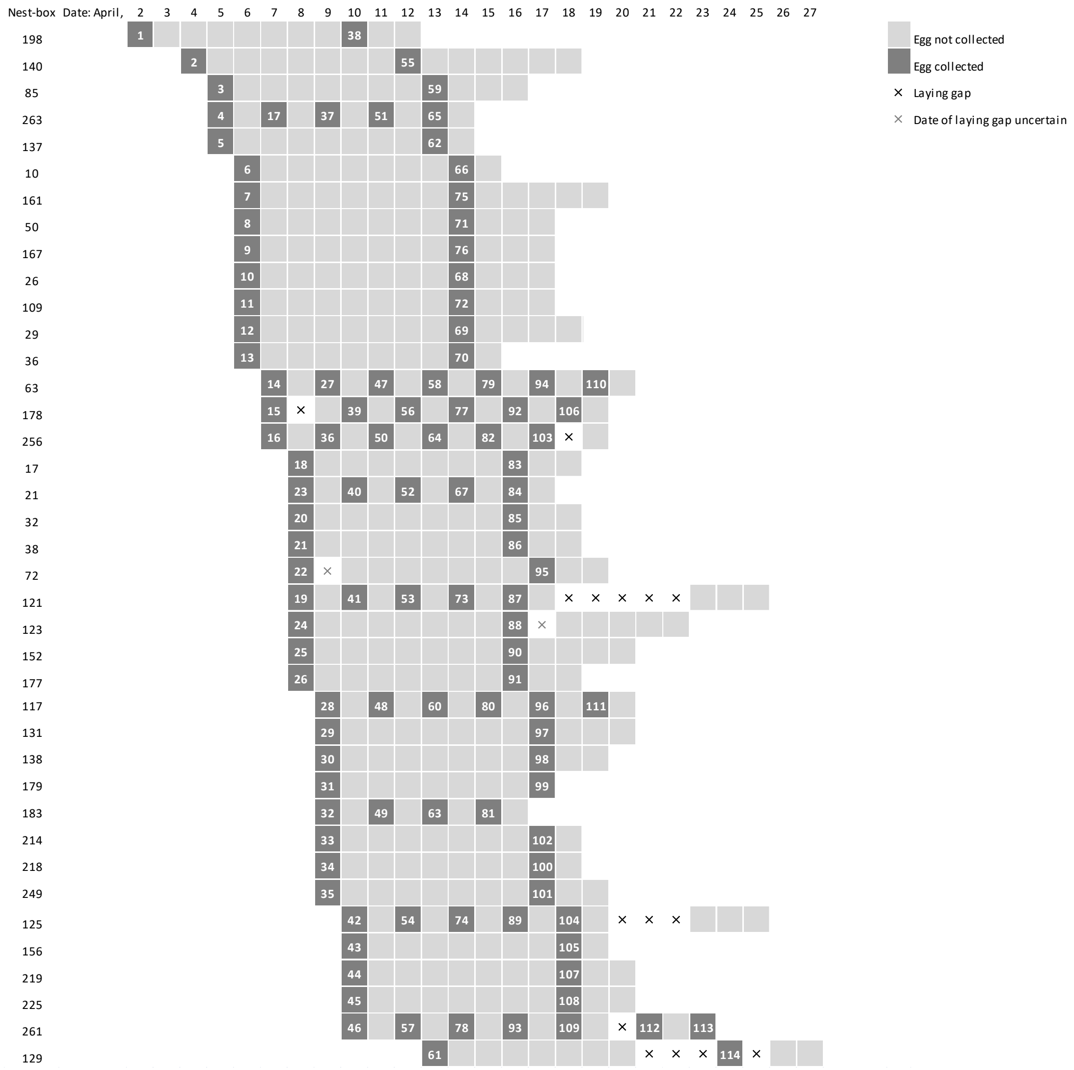

Supplementary Figure 3. Sampling scheme. The egg number inside the box reflects the order of collection.

1 Wilkin, T. A., Garant, D., Gosler, A. G. & Sheldon, B. C. Density effects on life-history traits in a wild population of the great tit *Parus major*: analyses of long-term data with GIS techniques. *Journal of Animal Ecology* **75**, 604-615 (2006).

2 Perrins, C. Population fluctuations and clutch-size in the Great Tit, *Parus major* L. *The Journal of Animal Ecology*, 601-647 (1965).

3 Van Noordwijk, A., McCleery, R. & Perrins, C. Selection for the timing of great tit breeding in relation to caterpillar growth and temperature. *Journal of Animal Ecology*, 451-458 (1995).

4 Perrins, C. Tits and their caterpillar food supply. *Ibis* **133**, 49-54 (1991).

5 Nilsson, J.-Å. & Svensson, E. Energy constraints and ultimate decisions during egg-laying in the blue tit. *Ecology*, 244-251 (1993).

6 Nilsson, J. Å. Time-dependent reproductive decisions in the blue tit. *Oikos* **88**, 351-361 (2000).

7 Ramsay, S. L. & Houston, D. C. Nutritional constraints on egg production in the blue tit: a supplementary feeding study. *Journal of Animal Ecology*, 649-657 (1997).

8 Ramsay, S. & Houston, D. The effect of dietary amino acid composition on egg production in blue tits. *Proceedings of the Royal Society of London B: Biological Sciences* **265**, 1401-1405 (1998).

9 Ojanen, M., Orell, M. & Väisänen, R. A. Role of heredity in egg size variation in the Great Tit *Parus major* and the Pied Flycatcher *Ficedula hypoleuca*. *Ornis Scandinavica*, 22-28 (1979).

10 Yom-Tov, Y. & Wright, J. Effect of heating nest boxes on egg laying in the blue tit (*Parus caeruleus*). *The Auk*, 95-99 (1993).

11 Perrins, C. & McCleery, R. Laying dates and clutch size in the great tit. *The Wilson Bulletin*, 236-253 (1989).

12 Boulinier, T. & Staszewski, V. Maternal transfer of antibodies: raising immuno-ecology issues. *Trends in Ecology & Evolution* **23**, 282-288 (2008).

13 Green, N. M. in *Advances in protein chemistry* Vol. 29 85-133 (Elsevier, 1975).

14 Board, R. & Fuller, R. Non-specific antimicrobial defences of the avian egg, embryo and neonate. *Biological Reviews* **49**, 15-49 (1974).

15 Shawkey, M. D. *et al.* Do birds differentially distribute antimicrobial proteins within clutches of eggs? *Behavioral Ecology* **19**, 920-927 (2008).

16 D'Alba, L. *et al.* Differential deposition of antimicrobial proteins in blue tit (*Cyanistes caeruleus*) clutches by laying order and male attractiveness. *Behavioral ecology and sociobiology* **64**, 1037-1045 (2010).

17 Wu, J. & Acero-Lopez, A. Ovotransferrin: structure, bioactivities, and preparation. *Food Research International* **46**, 480-487 (2012).

18 Jollès, P. & Jollès, J. What's new in lysozyme research? *Molecular and cellular biochemistry* **63**, 165-189 (1984).

19 Huang, H.-L. & Cheng, Y.-S. A novel minisequencing single-nucleotide polymorphism marker of the lysozyme gene detects high hatchability of Tsaiya ducks (*Anas platyrhynchos*). *Theriogenology* **82**, 1113-1120 (2014).

20 Boonyarittichaikij, R. *et al.* Mitigating the impact of microbial pressure on great (Parus major) and blue (Cyanistes caeruleus) tit hatching success through maternal immune investment. *PLOS ONE* **13**, e0204022 (2018).

21 Ruuskanen, S. *et al.* Geographical variation in egg mass and egg content in a passerine bird. *PLoS One* **6**, e25360 (2011).

22 Saino, N., Dall'Ara, P., Martinelli, R. & Møller, A. Early maternal effects and antibacterial immune factors in the eggs, nestlings and adults of the barn swallow. *Journal of Evolutionary Biology* **15**, 735-743 (2002).

23 Saino, N., Romano, M., Ambrosini, R., Ferrari, R. & Møller, A. Timing of reproduction and egg quality covary with temperature in the insectivorous Barn Swallow, *Hirundo rustica*. *Functional Ecology* **18**, 50-57 (2004).

24 Krištofík, J. *et al.* Do females invest more into eggs when males sing more attractively? Postmating sexual selection strategies in a monogamous reed passerine. *Ecology and evolution* **4**, 1328-1339 (2014).
25 Giraudeau, M. *et al.* Maternal investment of female mallards is influenced by male carotenoid-based coloration. *Proceedings of the Royal Society B: Biological Sciences*, rspb20101115 (2010).
26 Davison, F., Magor, K. E., Kaspers, B., Fred, D. & Karel, A. Structure and evolution of avian immunoglobulins. *Avian immunology* **1**, 107-127 (2008).
27 Kowalczyk, K., Daiss, J., Halpern, J. & Roth, T. Quantitation of maternal-fetal IgG transport in the chicken. *Immunology* **54**, 755 (1985).
28 Mann, K. & Mann, M. The chicken egg yolk plasma and granule proteomes. *Proteomics* **8**, 178-191 (2008).
29 Hargitai, R., Prechl, J. & Török, J. Maternal immunoglobulin concentration in collared flycatcher (Ficedula albicollis) eggs in relation to parental quality and laying order. *Functional Ecology* **20**, 829-838 (2006).
30 Morosinotto, C. *et al.* Predation risk affects the levels of maternal immune factors in avian eggs. *Journal of Avian Biology* **44**, 427-436 (2013).
31 Buechler, K., Fitze, P., Gottstein, B., Jacot, A. & Richner, H. Parasite-induced maternal response in a natural bird population. *Journal of Animal Ecology* **71**, 247-252 (2002).
32 Saino, N. *et al.* Early maternal effects mediated by immunity depend on sexual ornamentation of the male partner. *Proceedings of the Royal Society of London B: Biological Sciences* **269**, 1005-1009 (2002).
33 Pihlaja, M., Siitari, H. & Alatalo, R. V. Maternal antibodies in a wild altricial bird: effects on offspring immunity, growth and survival. *Journal of Animal Ecology* **75**, 1154-1164 (2006).
34 Surai, P., Speake, B. & Sparks, N. Carotenoids in avian nutrition and embryonic development. 1. Absorption, availability and levels in plasma and egg yolk. *The Journal of Poultry Science* **38**, 1-27 (2001).
35 Sillanpää, S., Salminen, J.-P., Lehikoinen, E., Toivonen, E. & Eeva, T. Carotenoids in a food chain along a pollution gradient. *Science of the Total Environment* **406**, 247-255 (2008).
36 Arnold, K. E., Ramsay, S. L., Henderson, L. & Larcombe, S. D. Seasonal variation in diet quality: antioxidants, invertebrates and blue tits *Cyanistes caeruleus*. *Biological Journal of the Linnean Society* **99**, 708-717 (2010).
37 Hõrak, P., Surai, P. F. & Moller, A. Fat-soluble antioxidants in the eggs of great tits *Parus major* in relation to breeding habitat and laying sequence. *Avian Science* **2**, 123-130 (2002).
38 Remes, V., Matysiokova, B. & Klejdus, B. Egg yolk antioxidant deposition as a function of parental ornamentation, age, and environment in great tits *Parus major*. *Journal of Avian Biology* **42**, 387-396, doi:10.1111/j.1600-048X.2011.05402.x (2011).
39 Szigeti, B. *et al.* Egg quality and parental ornamentation in the blue tit *Parus caeruleus*. *Journal of Avian Biology* **38**, 105-112 (2007).
40 Midamegbe, A. *et al.* Female blue tits with brighter yellow chests transfer more carotenoids to their eggs after an immune challenge. *Oecologia* **173**, 387-397 (2013).
41 Biard, C., Surai, P. F. & Moller, A. P. Effects of carotenoid availability during laying on reproduction in the blue tit. *Oecologia* **144**, 32-44, doi:10.1007/s00442-005-0048-x (2005).
42 Moller, A. *et al.* Carotenoid-dependent signals: indicators of foraging efficiency, immunocompetence or detoxification ability? *Poultry and Avian Biology Reviews* **11**, 137-160 (2000).
43 Koch, R. E. *et al.* No evidence that carotenoid pigments boost either immune or antioxidant defenses in a songbird. *Nature communications* **9**, 491 (2018).
44 Berthouly, A., Helfenstein, F., Tanner, M. & Richner, H. Sex-related effects of maternal egg investment on offspring in relation to carotenoid availability in the great tit. *Journal of Animal Ecology* **77**, 74-82, doi:10.1111/j.1365-2656.2007.01309.x (2008).
45 Biard, C., Surai, P. & Møller, A. An analysis of pre-and post-hatching maternal effects mediated by carotenoids in the blue tit. *Journal of evolutionary biology* **20**, 326-339 (2007).
